# Durability and Time-Dependent Properties of Low-Cement Concrete

**DOI:** 10.3390/ma13163583

**Published:** 2020-08-13

**Authors:** Keila Robalo, Eliana Soldado, Hugo Costa, Luís Carvalho, Ricardo do Carmo, Eduardo Júlio

**Affiliations:** 1 Department of Civil Engineering, Faculty of Sciences and Technology, University of Cape Verde, Praia 279, Cape Verde; keila.robalo@tecnico.ulisboa.pt; 2 Department of Civil Engineering, Architecture and Georesources, CERIS & Instituto Superior Técnico, University of Lisbon, 1049-001 Lisbon, Portugal; eduardo.julio@tecnico.ulisboa.pt; 3 Department of Civil Engineering, ISEC-Polytechnic Institute of Coimbra, 3030-199 Coimbra, Portugal; eacsoldado@gmail.com (E.S.); mr.luiscarvalho@gmail.com (L.C.); 4 Department of Civil Engineering, CERIS & ISEC-Polytechnic Institute of Coimbra, 3030-199 Coimbra, Portugal; carmo@isec.pt

**Keywords:** LCC, durability, carbonation resistance, creep, shrinkage, compactness, water/equivalent cement ratio

## Abstract

The sustainability concerns of concrete construction are focused both on the materials’ eco-efficiency and on the structures’ durability. The present work focuses on the characterization of low cement concrete (LCC), regarding time-dependent and durability properties. LCC studies which explore the influence of the formulation parameters, such as the W/C (water/cement ratio), W/Ceq, (which represents the mass ratio between water and equivalent cement), W/B (water/binder) ratio, and the reference curves, on the aforementioned properties are limited. Thus, several LCC mixtures were formulated considering two dosages of binder powder, 350 and 250 kg/m^3^, the former with very plastic consistency and the latter with dry consistency, which were combined with a large spectrum of cement replacement rates (up to 70%), through adding fly ash and limestone filler, and with different compactness levels. The main objectives were to study the influence of the formulation parameters on the properties: shrinkage and creep, accelerated carbonation and water absorption, by capillarity, and by immersion. The lifetime of structures produced with the studied LCC was estimated, considering the durability performance, regarding the carbonation effect on the possible corrosion of the steel reinforcement. LCC mixtures with reduced cement dosage and high compactness, despite the high W/C ratios, have low shrinkage and those with higher strength have reduced creep, however depending on W/C_eq_ ratio. Those mixtures can be formulated and produced presenting good performance regarding carbonation resistance and, consequently, a long lifetime, which is mandatory for a sustainable construction. LCC with 175 kg/m^3^ of cement dosage is an example with higher lifetime than current concrete with 250 kg/m^3^ of cement; depending on the XC exposure classes (corrosion induced by carbonation), the amount of cement can be reduced between 37.5% and 42%, since the LCC with 175 kg/m^3^ of cement allows reducing the concrete cover below the minimum recommended, ensuring simultaneously the required lifetime for current and special structures.

## 1. Introduction

Considering the challenge of climate changes, decarbonization must be an industrial priority in the construction sector, which has activities with a significant environmental impact, essentially due to the production of Portland cement. Current forecasts indicate an increase in cement consumption worldwide from the current annual value of around 4.2 billion tons to about 5.2 billion tons by 2050 [1]. With the current cement production technology, an average of 850 kg of CO_2_ emissions are released into the atmosphere for each ton of clinker produced [2]. Therefore, it is mandatory to develop new approaches allowing the production of structures using eco-efficient concrete with improved durability.

One of the solutions to mitigate the environmental impact associated with the production of the clinker, proposed by the European Cement Association (CEMBUREAU) in 2013 [3], is the use of additions as cement replacement in concrete, preferably by-products from other industries. With this measure, reductions of up to 3.5% of CO_2_ by 2030 and up to 8% by 2050 are expected. Associated with this measure, it is equally important to congregate the mechanical, time-dependent and durability properties, and the lifetime requirements of concrete structures. Currently, concerns about the durability and long-term performance of concrete are obstacles that limit the use of some additions in many applications. Water absorption, carbonation depth, shrinkage and creep performances are some of the parameters that can be used as indicators of durability and long-term performance of concrete.

During the design phase of a concrete structure, the service life is established, corresponding to the period during which it meets the requirements for safety, functionality, and aesthetics without unforeseen maintenance costs [4]. These requirements are influenced not only by the mechanical and time-dependent properties but also by the durability parameters. The durability of concrete is mostly directly linked to its ability to resist the penetration of gases and substances that are present in the environment. This ability depends mainly on the internal structure of the concrete, more on the binder matrix and less on the normal aggregates used, because these are usually inert and have reduced open porosity. Most reinforced concrete structures are usually designed to reach a service life of 50 and 100 years, mainly concerning the performance against corrosion of reinforcement steel.

Both durability and serviceability behaviors of reinforced concrete structures are influenced by time-dependent properties, mainly shrinkage and creep. Creep results from the viscoelastic behavior of concrete and corresponds to its deformation with age when subjected to constant stress level, which starts after the elastic deformation originated by loading. Nevertheless, it can be analyzed as basic creep (constitutive) and drying creep (depends on the concrete formulation, on curing conditions and on dimensions of the elements). Those values are fundamental to predict the total creep deformation. The increase of creep deformation is more accentuated soon after the load is applied and it reduces with increasing age, although it tends to stabilize generally before one year of age [5,6,7]. The creep deformations of concrete occur essentially on the hydrated cementitious binder matrix, in which the aggregates play an initial opposing role [5,7,8].

The type and dosage of cement and the water/binder ratio significantly affect the concrete creep [9], but that relation is not linear since creep depends on the amount of hydrated binder on the matrix. However, since those parameters, mainly the paste volume, have high influence on the strength of the concrete matrix [10], the concrete compressive strength at the age of load application is an important parameter, which indirectly includes the remainder [5,7]. The increase of creep is also influenced by the following parameters [5,6,11,12]: younger age of load application, since the matrix maturity is reduced; thermo-hygrometric curing conditions, especially higher temperature and lower relative humidity; reduction of the cross-section, since the area exposed to drying is higher. The creep prediction proposed by the main codes depends, directly or indirectly, on the mentioned factors. The evolution of the creep coefficient, φ (t, t_0_)-Equation (1), over age t is predicted by Eurocode 2 (EC2) [13] and by Model Code 2010 (MC10) [14], according to the following parameters: loading age (t_0_); type of cement; cross-sectional dimensions; compressive strength of concrete (f_cm_); and thermo-hygrometric conditions (relative humidity and temperature). The notional creep coefficient, φ0, is obtained from Equation (2) and considers those parameters, being the evolution curve obtained from Equation (3).

(1) $$\varphi \left({t,t}_{0}\right){=\varphi }_{0}\cdot \beta_{c}{(t,t}_{0})$$

(2) $$\varphi_{0}{=\varphi }_{\mathrm{RH}}\cdot {\beta (f}_{\mathrm{cm}})\cdot {\beta (t}_{0})$$

(3) $$\beta_{c}\left({t,t}_{0}\right)={\left[\frac{{(t-t}_{0})}{{(\beta }_{H}{+t-t}_{0})}\right]}^{0.3}$$

The shrinkage deformation, ε_cs_, or total shrinkage, is the dimensional variation mainly caused by the combined effects of the drying shrinkage, ε_cd_, and the autogenous shrinkage, ε_ca_. However, the sum of drying and autogenous shrinkage values is a simplification, since both parameters are caused by the reduction of the relative humidity (RH) of the concrete. Drying shrinkage occurs due to evaporation at the concrete’s surface, while autogenous deformation is caused by partial emptying of the gel pores as a consequence of cement hydration [15,16,17]. Nevertheless, for concrete with reduced cement dosage, the major component is drying shrinkage, since autogenous component is very low. According to EC2 [13] and MC10 [14], the evolution of ε_cd_ with age depends on the concrete strength, type of cement, geometry, curing conditions and initial age of drying. In EC2, the evolution of ε_ca_ with age depends only on the concrete strength, while in MC10 it depends on both concrete strength and cement type. The drying shrinkage component, ε_cd_ (t), can be estimated, according to EC2, using Equations (4)–(6). In these, the estimated drying shrinkage, ε_cd,0_, considers the most influent parameters mentioned above and β_ds_ (t, t_s_) defines the shape of the shrinkage evolution curve.

(4) $$\varepsilon_{\mathrm{cd}}\left(t\right){=\beta }_{\mathrm{ds}}{(t,\;t}_{s})\cdot k_{h}\cdot \varepsilon_{\mathrm{cd},0}$$

(5) $$\varepsilon_{\mathrm{cd},0}=0.85\;\left[(220+110\cdot \alpha_{\mathrm{ds}1})\cdot \exp\left({-\alpha }_{\mathrm{ds}2}\cdot \frac{f_{\mathrm{cm}}}{10}\right)\right]\cdot 10^{-6}\cdot \beta_{\mathrm{RH}}$$

(6) $$\beta_{\mathrm{ds}}\left(t,t_{s}\right)=\frac{\left(t-t_{s}\right)}{\left(t-t_{s}\right)+0.04\sqrt{h_{0}^{3}}}$$

Shrinkage of concrete must be characterized or reliably predicted because, if excessive, it causes undesired effects for reinforced and prestressed concrete elements, namely: (i) transversal cracking, due to the restriction of reinforcement, compromising strength and durability of structural elements; and (ii) excessive pre-stress losses, changing the structural behavior. Thus, the concrete formulation parameters must take into account the shrinkage minimization, in addition to complying with the other specifications, thereby contributing to guarantee the quality and durability of the concrete structures. The increase of concrete shrinkage is mainly influenced by factors such as: the increase of binders dosage and the hydration heat; the increase of water/binder ratio; the increase of the air volume in the paste and the permeability of the concrete; the worsening of thermo-hygrometric curing conditions (high temperatures and low relative humidity); the reduced age at the start of drying; the lower stiffness of the aggregates; and the reduction of cross-section dimensions [11,18,19].

In general, several supplementary cementitious materials can be used as additions to the binder paste of concrete, in a strategy of cement partial replacement, being the pozzolanic additions and ground granulated slags the most effective and promising additions to that purpose. Between the commonly used pozzolanic additions are silica fume, fly ash or metakaolin, among others, which generally improve concrete workability, strength and durability [20,21,22,23,24,25]. Silica fume has a high cost and is mainly used for concrete with high or ultra-high performances, with replacement dosages up to 10% in mass of binder. Metakaolin addition, despite having significant energy footprint and cost associated to its production, is eco-friendlier than Portland cement and it can also improve fresh and hardened properties when used in binary or ternary binder combination [26,27]. Nevertheless, fly ash addition is well known by its fineness and the spherical shape of its particles, which allows high water reductions and the increase of concrete packing density, resulting in significant performance improvements. When incorporated in the concrete matrix, fly ash promotes high workability increase, a reduction of hydration heat, an increase of maximum strength, a reduction of permeability, and a consequent improvement of durability [20,21,22,23,24,25]. Those advantages are associated with its water-reducing property, that is also related with the increase of mechanical properties and the reduction of drying and thermal shrinkage of the mixtures. When properly cured, fly ashes are capable of providing very reduced permeability and improved durability [20]. On the other hand, high concentrations of natural radionuclides may occur on pozzolanic cements and slag cements [28]. It is known that some cements used in the construction industry of several regions contain natural radioactive isotopes, such as radium (^226^Ra), thorium (^232^Th) and potassium (^40^K), and their specific activity may exceed the average specific activity [28]. The addition of fly ash to Portland cement can have an effect on the highest concentrations of natural radionuclides. Consequently, it is prudent to control the fly ash addition into cementitious matrix, by limiting its proportion.

Accordingly, the present work has as main objectives: (i) to produce low-cement concrete (LCC) mixtures varying the main composition parameters, namely, the compactness and the water/binder ratio, the cement dosage with partial replacement by combined additions and the granulometric reference curve; (ii) to relate the cement dosage and the mechanical strength of concrete, as well as other formulation parameters, with the evolution and amplitude of the shrinkage and creep; (iii) to evaluate the influence of the compactness of the mixtures, and also W/C and W/B ratios, on the development of shrinkage and creep, as well as on other properties of concrete related to durability; (iv) to analyze the influence of the reference curves, Faury and Alfred, on concrete creep; (v) to relate the formulation parameters of the mixtures with the water absorption of concrete, by immersion and by capillarity, as well as with the carbonation depth; (vi) to estimate the lifetime of structures produced with the characterized LCC, taking into account the resistance to carbonation, which is associated with the corrosion risk of the steel reinforcement; (vii) to compare the results obtained with the standards and study possible improvements.

## 2. Research Significance

Recently, several researchers have investigated the individual effect of the high volume of fly ash as a partial replacement of Portland cement, and have objectively proved that these additions have a significant influence on the concrete properties [20,21,22,23,24,29,30,31,32]. However, studies on the effects of a high volume of combined fly ash and limestone filler contents on concrete are scarce and limited, mainly when formulated with reduced cement content. Thus, it is necessary to carry out studies that simultaneously use high volumes of the two additions as partial replacements of Portland cement, in order to maximize the environmental and economic benefits in the production of concrete. Based on this combination, several concretes with LCC were developed taking advantage of the proven benefits of granulometric optimization and use of high strength cement and superplasticizer [29,30,33,34]. These concrete mixtures were characterized in a previous study in terms of mechanical strength and workability [33]. It has been proved that the combination of the two additions, fly ash and limestone filler, can minimize some limitations of the single used of fly ash use: concrete with high content of fly ash can present a slow hardening at young age [33]; high volumes of fly ash can increase the radionuclides [28]. The idea of combining those is due to the reduced cost and easy availability of limestone filler, combined with good filling effect and low environmental impact [35]. This filler addition does not participate in the chemical reaction but acts as a setting accelerator during the initial hydration of the cement; in the cement hydration process, the limestone filler causes its main constituent, calcium carbonate (CaCO3), to modify the initial reaction of tricalcium aluminate (C3A), accelerating the hydration rate of tricalcium silicate (C3S) and decreasing the hardening times [36,37,38,39]. The replacement of cement with fly ash has also been a preferred option for LCC, in which high dosages were used in the formulations and, therefore, the cement dosages were well below those recommended by the EN 206 [40]. Thus, it is highly pertinent to deepen the studies of this concrete to understand all its properties and, as a result, to encourage its use.

The granulometric optimization and the use of superplasticizer also contribute to the improvement of the efficiency of the binder paste and the durability and strength of the concrete [30,34,41], as they allow the compactness of the mixture to be increased and, consequently, to reduce the need for water and binder content, since there is less space available between the particles, making it a denser and less permeable matrix.

Problems with durability of concrete are generally associated with the mentioned parameters, generally in matrices with high porosity and excess of water [20], providing reduced resistance to carbonation and consequently a precocious reinforcement corrosion. Several studies address durability in fly-ash and eco-friendly concretes, regarding resistance to carbonation, chlorides migration, permeability, and porosity [42,43,44,45,46,47]. The latter, characterized by the immersion test, although not allowing to directly estimate the durability of the concrete, as the other mentioned tests, it gives an indication of the volume of pores [47]. However, the bibliography is inexistent regarding the durability of concrete with low-cement dosage and high compactness, combined with fly ash and limestone filler additions.

The use of different granulometric curves in the optimization affects the relative proportions of the aggregates used, which in turn influence the compactness, the workability, and the Young’s modulus [48]. Regarding the latter, the influence is higher for smaller cement dosages [33] and in relation to compressive strength, these tend to be less relevant with age, since the binder paste acquires greater importance in strength over time [33]. The different granulometric curves and the variation of compactness also have a strong influence on the analysis of shrinkage and creep, since those are parameters that strongly depend on the material constituents of the concrete. The analysis of shrinkage and creep in LCC is certainly different from that of current concrete and its different formulation affects most of the influencing parameters to those properties, mainly those that are related with the binder matrix. Also the predictions presented by the main codes, EC2 [13] and MC10 [14], are certainly inadequate, since they were developed for concrete with current formulation. The study of these concrete properties is less common, probably due to time-consuming and to specific equipment required, which combined with recent developments of LCC formulation, make the related bibliography very scarce or inexistent.

## 3. Experimental Program

### 3.1. Concrete Formulation

In order to evaluate the formulation parameters of the binder paste and the efficiency of granulometric optimization in the mechanical performance of concrete with low-cement content, Costa et al. [33] and Freitas et al. [49] developed LCC mixtures, which were characterized herein in terms of time dependent and durability properties. Those concretes contain in their composition: cement CEM I 52.5R (C); additions of class F fly ash (FA) and limestone filler (Lf); superplasticizer MasterGlenium SKY 526 (SKY); water; fine siliceous sand 0/3 mm (S0/3); medium siliceous sand 0/4 mm (S0/4); rolled siliceous gravel 4/8 mm (G4/8) and crushed limestone gravel 6/14mm (C6/14). The proportion of these constituents was defined based on the methodology proposed by Lourenço et al. [50], and later modified by Costa et al. [7], which is initially based on the definition of the parameters required for the performance of the binder matrix and on the method of reference granulometric curves to obtain the aggregates’ proportions. The formulation parameters were adopted in order to minimize the cement dosage, having as main variations: the cement dosage and the replacement by additions; the amount of binder powder in the paste; the compactness and the water/binder ratio (W/B) and; the granulometric curves. Thus, two series were considered with different dosages of binder powder in the matrix and with different workability: (i) series C, with a current dosage (350 kg/m^3^), with a target consistency of class S3 (very plastic consistency); (ii) low-cement (LC) series, with a reduced dosage (250 kg/m^3^), varying the effective cement dosage in substitution regime with the addition of limestone filler and fly ash, with a dry consistency and low cohesion.

The considered compactness was in general 0.86 in LC concrete and 0.81 in C concrete, with the corresponding W/B ratios being 0.47 and 0.48, respectively. In both series, when varying the cement dosage (75, 125, 175 and 250 kg/m^3^), the water/cement (W/C) ratio consequently varies. The substitution of cement by additions was defined giving priority to the filler until a maximum equal to the cement mass was reached, followed by the complement of fly ash, when necessary, until the dosage for the binder powder was reached. A subseries of C concrete was also formulated, named C_B, varying the compactness values from 0.81 in C series to 0.835 and to 0.855 in mixtures C200B and C175B (Table 1). Once the parameters of the binding matrix were defined, the methods of the Faury’s granulometric curve [48,50] and the Alfred’s curve [51], being the latter developed by Funk and Dinger [52], were used to optimize the proportions of the aggregates, thus varying the aggregates proportions [50].

The formulated mixtures were referenced considering the amount of cement (75, 125, 175, 200, 250 kg/m^3^) and the amount of binder powder (having 250 kg/m^3^ in LC concrete and 350 kg/m^3^ in C concrete). In the LC series, formulation by the Alfred’s curve (mixtures LC75, LC125, LC175 and LC250) was considered. The other series or subseries (LC_F, C and C_B) were formulated using the Faury’s reference curve. In LC250 mixture, a compactness variation, between 0.86 and 0.80, and consequently the W/B ratio were also considered for LC mixture with 250 kg of cement dosage (resulting in LC250 and LC250A mixtures). The dosages of all constituents mentioned are shown in Table 1.

### 3.2. Workability and Mechanical Properties of Concrete

The characterization in fresh state and the mechanical properties of the LCC mixtures were carried out by Costa et al. [33] and Freitas et al. [49] and are presented in Table 2. In a fresh state, the density was evaluated, in order to control the formulation parameters and workability was characterized through the slump-test and the degree of compactability (D_Comp), following the standards EN 12350-2 [53] and EN 12350-4 [54]. The latter was performed for LC mixes with Alfred’s curve, since they were not validated in the consistency test, collapsing due to lack of cohesion.

The determination of the compressive strength, was carried out according to EN 12390-3 [55], in cubic specimens with 150 mm edge. The results correspond to the mean values of test in three samples, for each considered age (7, 28 and 56 days), resulting f_cm7,_ f_cm28_, f_cm56_ (compressive strength measured at 7, 28 and 56 days respectively). At 28 days of age, the following properties were also characterized: splitting tensile strength, f_ctm,sp_, according to EN 12390-6 [56] in three prismatic test specimens of 100 × 100 × 200 mm^3^; flexural strength, f_ctm,f_, following the standard EN 12390-5 [57] in two prismatic test pieces of 100 × 100 × 400 mm^3^; the Young’s Modulus, E_cm_, according to the specification of National Laboratory of Civil Engineering (LNEC) E-397 [58] in two prismatic specimens of 100 × 100 × 400 mm^3^. The average values of that properties are shown in Table 2.

### 3.3. Durability and Time-Dependent Tests

**Figure 1:**
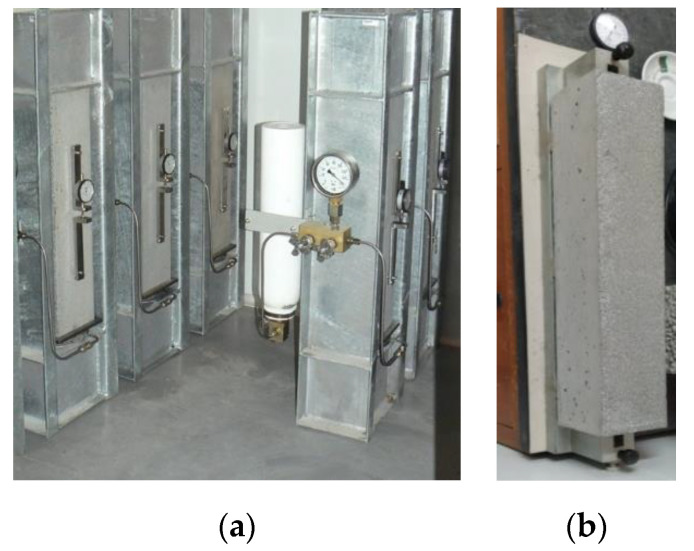
Compressive creep test (**a**) and total shrinkage test (**b**).

After the formulation and mechanical characterization of concretes with low dosage of cement, the study comprised the characterization of time dependent and durability properties. Regarding time dependent properties, creep was characterized experimentally through the compression creep test [59] using 100 × 100 × 400 mm^3^ prismatic specimens (Figure 1), and the total shrinkage, ε_cs,_ (Figure 1), was characterized according to the specification LNEC E398 [60], testing two twin prismatic specimens of 100 × 100 × 500 mm^3^ for each concrete.

**Figure 2:**
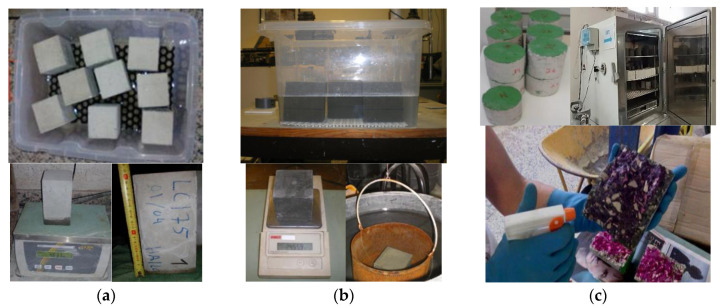
Durability tests: (**a**) water absorption through capillarity; (**b**) water absorption by immersion; (**c**) accelerated carbonation test.

The durability performance was characterized by the following tests (Figure 2): (i) water absorption by capillarity, according to LNEC E393 specification [61], using three 100 × 100 × 200 mm^3^ prismatic specimens; (ii) water absorption by immersion, which was carried out on cubic specimens with 100 mm edge and according to LNEC E394 specification [62]; and (iii) accelerated carbonation test, carried out following the LNEC E391 specification [63], using two cylindrical samples with 100 mm diameter and 50 mm height, for each considered exposure period (7, 14, 28, and 42 days).

## 4. Results and Discussion

### 4.1. Shrinkage

**Figure 3:**
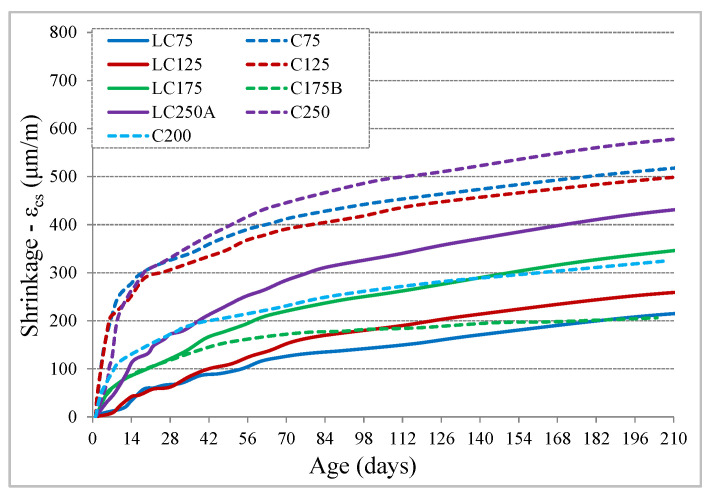
Evolution of total shrinkage with age.

The evolution of the total shrinkage, ε_cs_, measured in the test specimens of each mixture, is shown in Figure 3. The LC series concretes (with 250 kg of binder powder) have developed generally reduced shrinkage values, between 215 and 430 μm/m at 210 days, although they tend to increase with cement dosage increase. Concretes of C series (with 350 kg of binder powder) have values between 500 and 570 μm/m at 210 days, in concrete with a compactness of 0.81 and tends to gradually reduce with compactness increase, in C200 and C175B concretes, although having similar value of equivalent cement, their compactness is 0.84 and 0.86, respectively.

**Figure 4:**
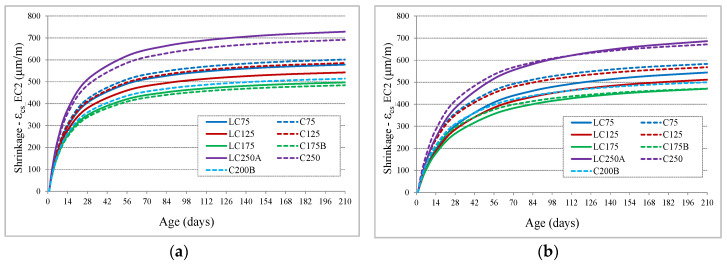
Comparison of the evolution of shrinkage by Eurocode 2 (EC2): (**a**) prediction curve with EC2 equations; (**b**) adaptation of the experimental evolution to prediction curve using the β_shape_ coefficient.

Regarding the shape of the shrinkage curve, compared with the EC2 equations, it was found that there is an inadequacy with the prediction. While the experimental evolution is slower after drying begins, particularly in the LC series, and continues to evolve significantly after 90 days, the prediction curves show a more accelerated evolution at a younger age, tending to stabilize earlier (Figure 4a) than experimental results. The expected shrinkage values of EC2 at 210 days of age correspond to approximately 94% of the value at infinite time. Taking into account that the shape of the curve depends on the coefficient β_ds_ (t,t_0_), Equation (6), the value of the second part of the denominator was adjusted, from 0.04 to a β_shape_ coefficient, Equation (7), which can assume values of 0.06 for concretes of C series and 0.08 for concretes of LC series, allowing a better adaptation of the evolution with age (Figure 4b), in comparison to experimental results. With this adjustment, the shrinkage values predicted at 210 days become about 88% to 91% of the value at infinite time. It should be noted that the autogenous shrinkage component is very low in this type of concrete, being the prediction less than 10% in all concrete and less than 5% in most mixtures.

(7) $$\beta_{\mathrm{ds}}\left({t,t}_{s}\right)=\frac{{(t-t}_{s})}{\left({t-t}_{s}\right)+\beta_{\mathrm{shape}}\sqrt{h_{0}^{3}}}$$

**Figure 5:**
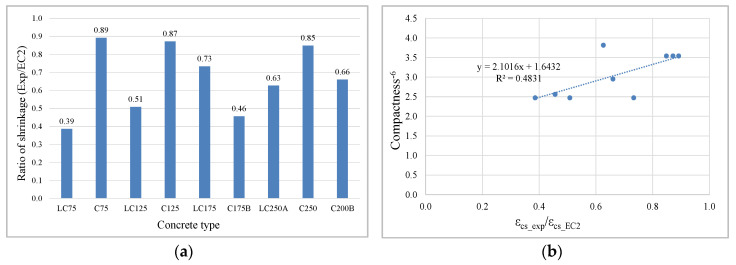
Influence of compactness on ratio of shrinkage Exp/EC2: (**a**) ratio for each concrete; (**b**) correlation between the ratio and compactness^−6^.

Regarding the shrinkage amplitude there is a significant difference between prediction and experimental values in several concretes, explained by the shrinkage ratios (Exp/EC2), Figure 5a, which are more significant in LC with lower cement content and in C175B concrete with high compactness. Concrete with 350 kg of powder content (C series) and current compactness are the ones that best match the EC2 prediction range. An evident influence of the increased compactness on shrinkage reduction was identified, the best obtained correlation being between that ratio and the compactness^−6^. The dispersion of two concretes to that correlation (Figure 5b) suggests that there are even other parameters influencing the shrinkage amplitude.

### 4.2. Creep

**Figure 6:**
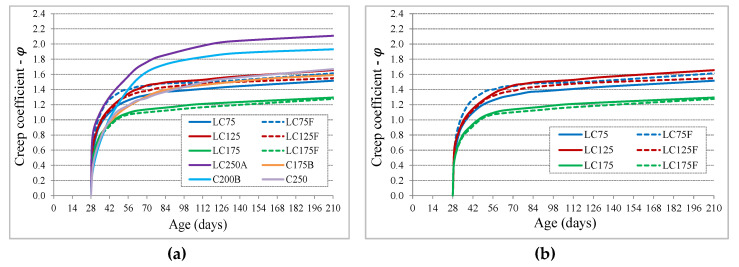
Evolution with age of creep coefficient: (**a**) all concretes; (**b**) concretes LC with different granulometric curves (LC-Funk and Dinger; LC_F-Faury).

The results obtained for the creep characterization are presented through the evolution, over time, of the creep coefficient, φ_c_(t) (Figure 6), with the load having been applied at 28 days of age to assure a proper maturity of concrete. Since an early loading age promotes higher creep deformation for concrete in general, for this type of concrete it is advisable that the load occur at minimum age of 28 days, considering the lower evolution of strength and maturity of these concretes. The creep was evaluated in all concretes, including the LC and LC_F series (with variation of the granulometric curve), Figure 6b, with only C75 and C125 concretes being excluded since they have reduced mechanical performance for structural purposes. Regarding the influence of the granulometric curve, used in the formulation of LC concrete, on concrete creep, no apparent influence was noticed. The concretes with lower equivalent cement dosage (LC75 and LC125, with C_eq_ of 85 and 125, respectively) have similar evolutions and amplitudes, with creep coefficients close to 1.6 at 210 days of age. LC175 concrete has a smaller creep amplitude compared to those mentioned (creep coefficient close to 1.3 at 210 days of age), as expected, because it has a higher cement dosage and greater strength. Despite the reduced influence of the granulometric curve is proven in creep coefficient, the creep deformation is lower in concrete with Alfred’s curve, since the Young’s modulus of those concretes (LC75, LC125 and LC175) are 11% to 30% higher than those formulated by the Faury’s curve (LC75F, LC125F and LC175F). That difference is higher in concretes with a lower cement dosage and decreases with the increase of its dosage. With the exception of LC250A concrete (concrete with high W/B ratio and reduced compactness, 0.80) and C200 (concrete with compactness of 0.84), which showed a creep coefficient at 210 days, from 1.92 to 2.12, respectively. The remaining concretes show a similar evolution and amplitude, whose coefficient is around 1.6 at 210 days. Part of the influence of these values is evidenced by the different mechanical strengths, as mentioned.

**Figure 7:**
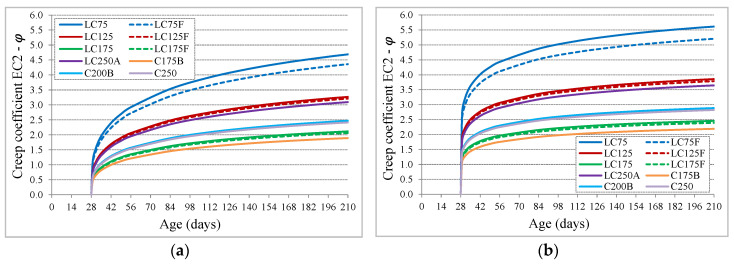
Predicted evolution with age of creep coefficient (EC2): (**a**) curves with αt = 0.30; (**b**) curves with αt = 0.15.

Comparing the shape of evolution creep curve with the EC2 prediction, which is the same as fib MC10, a high divergence was found. While the experimental evolution is quite accelerated after load application and attenuates quickly about 28 days after load loading, the prediction curves show a slower evolution (Figure 7a). The creep coefficient values predicted at 210 days correspond to about 70% to 74% of the value at infinite time. Considering that the shape of the creep curve depends on the coefficient β_c_ (t,t_0_), Equation (3), the exponent of that ratio, α_t_, was adjusted from 0.30 to 0.15, Equation (8), obtaining a better adaptation of the evolution with age (Figure 7b). With this adjustment, the values of the creep coefficient, predicted at 210 days of age, become about 84% to 87% of the value at infinite time.

(8) $$\beta_{c}\left({t,t}_{0}\right)={\left[\frac{{(t-t}_{0})}{{(\beta }_{H}{+t-t}_{0})}\right]}^{\alpha_{t}}$$

**Figure 8:**
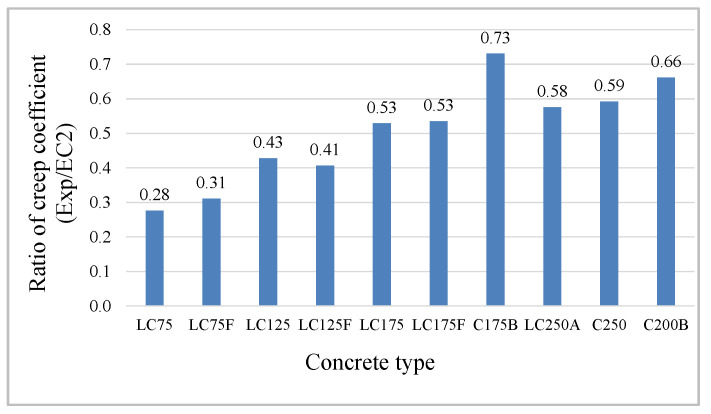
Ratio between creep coefficients Exp/EC2.

**Figure 9:**
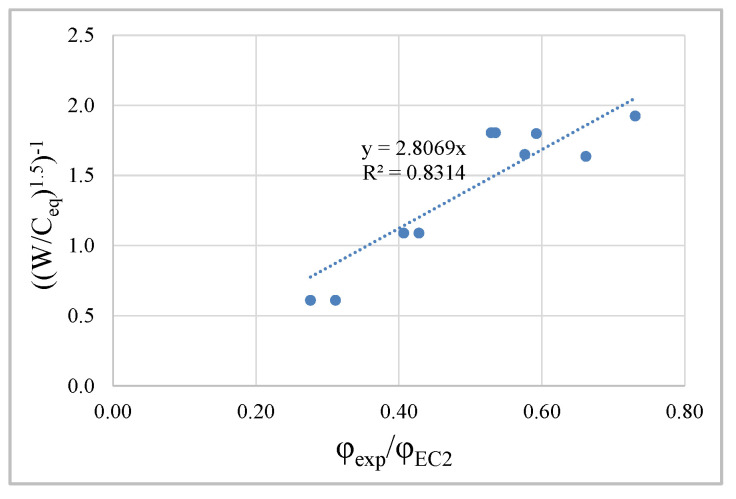
Correlation between W/Ceq ratio and Exp/EC2 relationship of creep coefficients.

Regarding the amplitude, there is a huge difference between the prediction and the experimental values, translated by the ratios between the creep coefficients (Exp/EC2), Figure 8, being higher in LC concrete with lower cement dosage and in C concrete with less compactness and higher W/B ratio. In this sense, the best correlation between this ratio and the concrete formulation parameters was studied, and it was found that the ratio (W/C_eq_), which represents the mass ratio between water and equivalent cement, presents the best fit (Figure 9).

Knowing that the amplitude of the creep curves strongly depends on the β(f_cm_) coefficient, Equation (9), the result of the correlation obtained was incorporated in determining this coefficient. Due to the verified dispersion, the amplitude of the correlation was reduced in order to ensure conservative predictions, resulting in a new proposal to predict coefficient β(f_cm_), expressed by Equations (10) and (11).

(9) $$\beta \left(f_{\mathrm{cm}}\right)=\frac{16.8}{\sqrt{f_{\mathrm{cm}}}}$$

(10) $$\beta \left(f_{\mathrm{cm}}\right)=\frac{16.8}{\beta_{\mathrm{Ceq}}\times \sqrt{f_{\mathrm{cm}}}}$$

(11) $$\beta_{\mathrm{Ceq}}=2.5\times {\left(W/\mathrm{Ceq}\right)}^{1.5}$$

**Figure 10:**
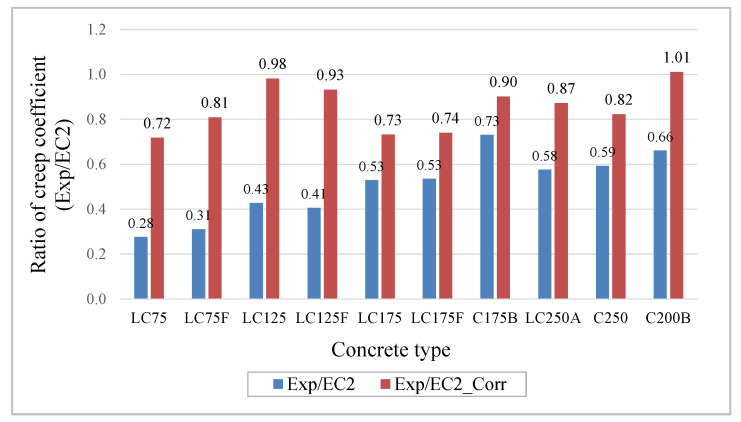
Ratio of creep coefficients: Exp/EC2 and Exp/EC2_Corr.

With this correction, the ratios between the creep coefficients (Exp/EC2_Corr), Figure 10, show a significant improvement in prediction, being this conservative, since some experimental results are up to about 28% below the corrected prediction, although with much better approximation to the experimental characterized behavior.

**Figure 11:**
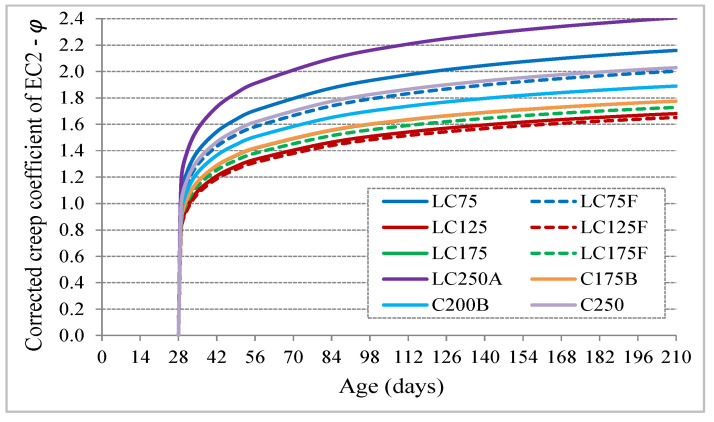
Evolution with age of corrected creep coefficient of EC2.

With this new proposal to predict the creep coefficient, including the correction of the curve shape and its amplitude, which depends on the W/C_eq_ ratio, the creep curves of the characterized concrete have much better adjustment compared to experimental results (Figure 11).

### 4.3. Water Absorption by Immersion

**Figure 12:**
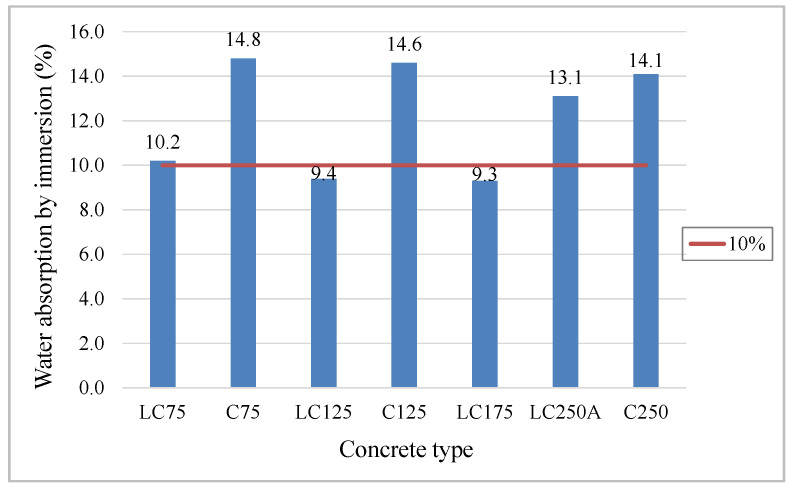
Average values of water absorption by immersion.

The results of the water absorption test by immersion at atmospheric pressure are plotted in Figure 12. Through the analysis of the graph, it was found that LC concrete, with a compactness of 0.86, absorb much less water when compared to the concrete of the C series, with compactness of 0.81 (Figure 12). Compactness has a high influence on water absorption, as it increases, the volume of water and air reduces, resulting in denser and less permeable matrices, therefore less susceptible to absorption. Neville [6] defines that a good quality concrete has absorption by immersion below 10%. This study reveals that the mixtures of LC series, in addition to registering absorption values slightly below 10%, present approximately less 5% of water absorption (about 1/3) when compared to the C series. LC250A and C series mixtures are above 10%, locating between 13% and 15%, thus with higher porosity and more susceptibility to water absorption. Despite both w/c ratio and paste volume have major effects on the porosity, permeability and strength [64,65], the higher compactness of LC mixtures, and consequently the lower W/C ratio, revealed major influence on reducing the water absorption for these formulations, in comparison to the lower paste volume.

**Figure 13:**
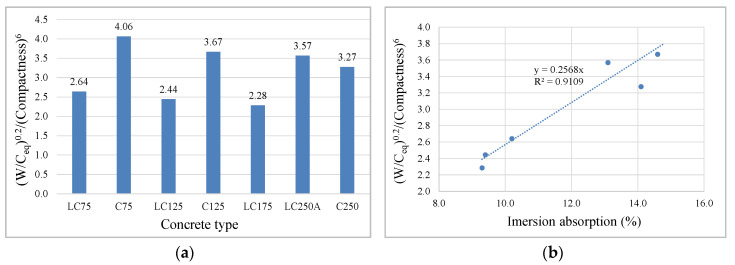
Relation between W/C_eq_ (**a**) and compactness depending on water absorption by immersion (**b**).

Furthermore, in each series, C and LC, it is clear that the water/equivalent-cement ratio, W/C_eq_ also has an important influence, albeit less than the previous parameter, since as the equivalent cement increases, this ratio decreases, and the calcium silicate hydrates (CSH) gel resulting from hydrated cement occupies the voids inside the matrix, also reducing absorption, because the matrix is more dense and less porous. The ratio that incorporates compactness and W/C_eq_ was studied, which provides the best correlation, and this relation is shown in Figure 13a. The high correlation between immersion absorption and this relation is shown in Figure 13b.

### 4.4. Capillary Water Absorption

**Figure 14:**
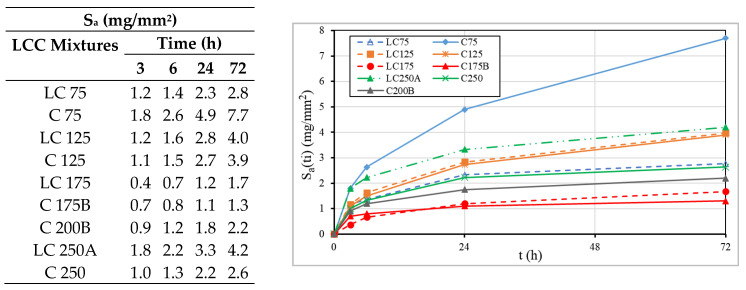
Average values of water absorption by capillarity at 3, 6, 24, and 72 h.

The capillarity test allows the determination of the amount of water that concrete absorbs by suction, through small pores. Figure 14 presents the average capillary absorption at the instant t_i_, Sa(t_i_), in mg/mm^2^.

In both series, the water absorption by capillarity occurs with greater intensity in the first 24 h and tends to stabilize over time, which occurs earlier for concretes with increased compactness and higher cement dosage. As the water starts to penetrate the concrete by capillarity, it is noticed that after the saturation of the pores at the lowest level of the specimen, the water level tends to rise. The maximum height that the water can reach is limited by the pressure difference between the free surface of the water outside the concrete and its surface in the capillary pores of the concrete, and also by the gravitational action. Hence the tendency for water absorption by capillarity to stabilize over time [4].

In C75 concrete there is also a pronounced initial evolution, but with an increasing trend until 72 h and with much higher values, at least 45% at 24 h and 80% at 72 h, comparatively with the other concretes. This must be associated with the reduced compactness combined with very low cement dosage, despite the high content of fly ash (FA/C = 2.7). When comparing this concrete with the others, it is noted that LC250A succeeds it with high capillary absorption, presenting a behavior with similar tendency, however less pronounced. Despite the cement dosage of 250 kg/m^3^, concrete LC250A have reduced compactness of 0.8, being the main reason for high capillary absorption.

When comparing results of C75 and LC75, it is noted that the latter presents lower values of capillary absorption than the C75, from 35% at 3 h to 65% at 72 h, increasing the difference with the duration of the test. Those differences are essentially due to greater compactness of the LC75, given that both concretes contain the same amount of cement and a high content of fly ash (1.3 times the proportion of cement in LC75 and 2.7 in C75). In these mixtures, it appears that the high fly ash content did not have much impact on the difference between the results of both concretes, otherwise, the differences could be smaller. This must be associated with low cement dosage, since the fly ash forms the gel of calcium silicate hydrates crystals (CSH), which improve the performance of the concrete, from the reaction with released hydrated lime (CH) during the clinker hydration [66]. As there is a small amount of the cement and consequently the hydrated lime, it limits the chemical activation of fly ash, proving that high fly ash content is effective when the cement dosage is not very reduced.

The previous comparison did not allow assessment of the influence of fly ash on capillarity absorption results, since both concretes had high FA/cement ratio, but very different compactness. However, when comparing LC75 with LC125, both with the same compactness, it is noted that the former shows lower capillary absorption than the LC125, from 18% in lower test duration to 43% for higher duration, also increasing the differences with the duration of the test. This difference proves the effectiveness of fly ash in LC75 concrete, also emphasizing that the LC125 has a cement dosage about 67% higher than that of the LC75 (Table 1), but similar values of equivalent cement. It is also verified that C125 and LC125 concretes have practically the same capillary absorption. The compactness of C125 concrete is 0.81 and it has 100 kg/m^3^ of fly ash in the paste, while the compactness of LC125 concrete is 0.86 but does not contain fly ash. Due to the significant difference between the compactness of the two concretes, it would be expected that the difference between the capillarity absorption values would be significant. This situation did not occur, thanks to the presence of fly ash that allows the reduction of absorption by capillarity.

The C175B and LC175 concretes show similar and better capillarity water absorption results than the other concretes, however LC175 concrete shows slightly lower results in the first 24 h, and from that period the trend was reversed, with the concrete C175B having inferior absorption. Considering that both concretes have the same amount of cement, 175 kg/m^3^, and approximately equal compactness, however differing on powder content (250 kg/m^3^ on LC175 and 350 kg/m^3^ on C175B) and on fly ash content (C175B contains 75 kg/m^3^ of fly ash). The combination of fly ash with cement may be associated with that improvement in C175B binder paste, because this addition allows the development of new compounds that fill the pores of concrete matrix.

**Figure 15:**
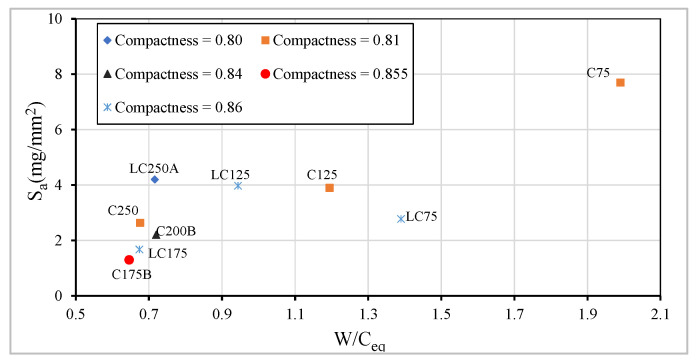
Relation between the capillarity water absorption vs. W/C_eq_.

The differences found in the different formulated concrete can be seen from the analysis of Figure 15, in which the relation between parameters such as compactness, the water/equivalent-cement ratio, equivalent-cement ratio determined according to EN 206 [40], and the absorption of water by capillarity is presented. The plotted graph highlights the importance of controlling compactness as well as the water/equivalent-cement ratio in LCC. For this type of concrete, the capillary absorption tends to increase with the cement dosage decrease, but it can be compensated with the increase of fly ash dosage, thus obtaining good results in terms of this durability indicator. For concrete in general, it is evident that increasing compactness has an important role on capillary reduction.

**Figure 16:**
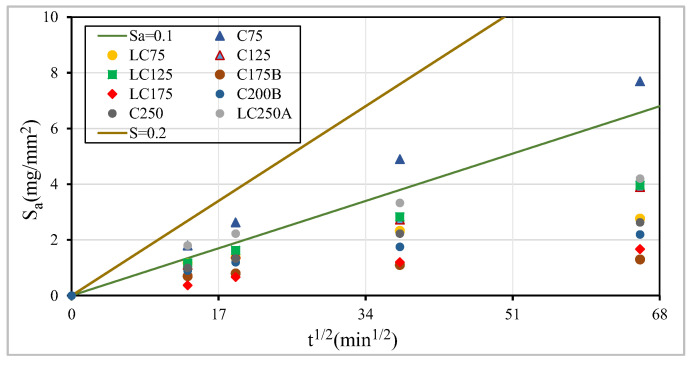
Capillarity water absorption through square root of time.

Figure 16 shows the mean values of absorption by capillarity, S_a_ (in milligrams per square millimeter) as a function of the square root of time, obtained for each mixture, as well as the slope that expresses the capillary absorption coefficient of the concrete. According to Browne’s classification cited in Coutinho [4]: high quality for concretes whose capillary absorption coefficients, S_a,_ are less than 0.1 mg/(mm^2^·min^0.5^); medium quality for concretes whose coefficients are between 0.1 e 0.2 mg/(mm^2^·min^0.5^). It can be seen that C75 concrete can be classified as medium quality, C250 starts with medium quality and obtains a better classification over time, while the rests are classified as high quality.

The results of capillarity absorption allow us to conclude that just increasing the cement dosage does not imply an improvement in the durability characteristics of the concrete, clearly demonstrating the importance of packing optimization and of binder paste, as its impact is significant on the compactness of the concrete [33].

### 4.5. Carbonation

**Figure 17:**
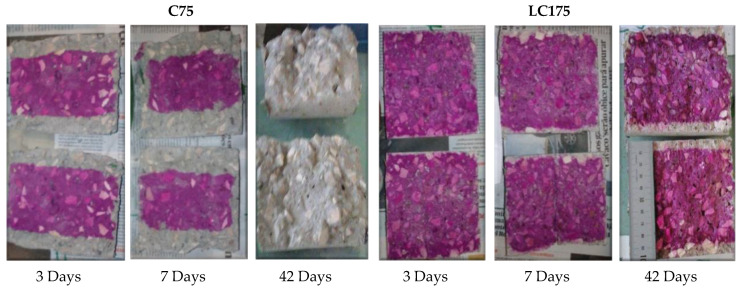
Concrete specimens C75 and LC175 with phenolphthalein after 3, 7 and 42 days of exposure to CO_2_.

The concrete carbonation is associated with the penetration, by diffusion, of carbon dioxide into the concrete. When it is dissolved in concrete, it induces chemical reactions to dissolve the crystalline phases of concrete, such as calcium hydroxide (CH) giving rise to the production of carbonates, such as calcium carbonate, which leads to a reduction of concrete pH [19,67]. Therefore, the pH of concrete gives information about this durability parameter. Thus, a solution of phenolphthalein was vaporized on the concrete exposed to carbon dioxide, and the areas where the pH was higher than 8 show a crimson color, whereas the areas where the concrete was carbonated, the alcoholic solution of phenolphthalein remained colorless (Figure 17).

**Figure 18:**
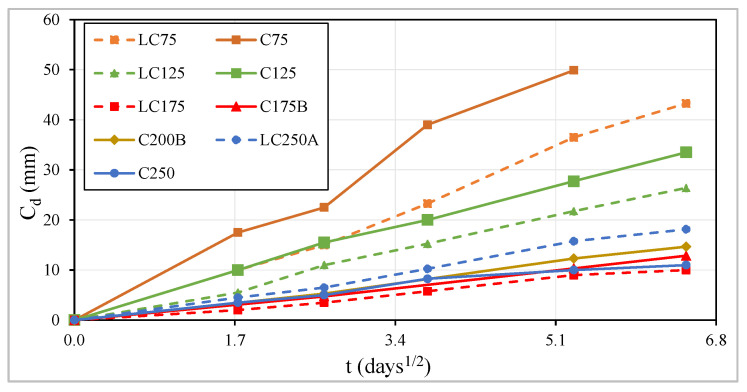
Carbonation depth vs. square root of time.

Figure 18 and Table 3 show the average carbonation depth values, C_d_, in millimeters. Looking at the carbonation development curves along the square root of time (Figure 18), it appears that the carbonation speed, in most of the characterized concrete, is practically constant over square root of time. For a linear time scale, the speed is higher in the first days and tends to be slower over time, because after carbonation, the resulting products occupy the empty spaces in the pores of the concrete matrix and hinder the diffusion of carbon dioxide in the concrete, decreasing thus the carbonation rate.

**Figure 19:**
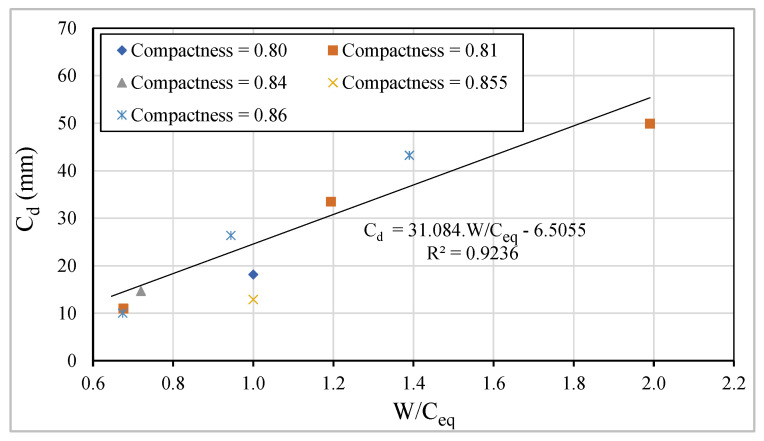
Relationship between C_d_ vs. W/C_eq_.

In each series, i.e., maintaining the amount of binder powder, it is proved that the carbonation depth decreases with the increase in the amount of cement or equivalent-cement. Figure 19 shows a linear correlation between C_d_ after 42 days of exposure to carbon dioxide and W/C_eq_, whose correlation coefficient, R^2^, is approximately 0.92.

**Figure 20:**
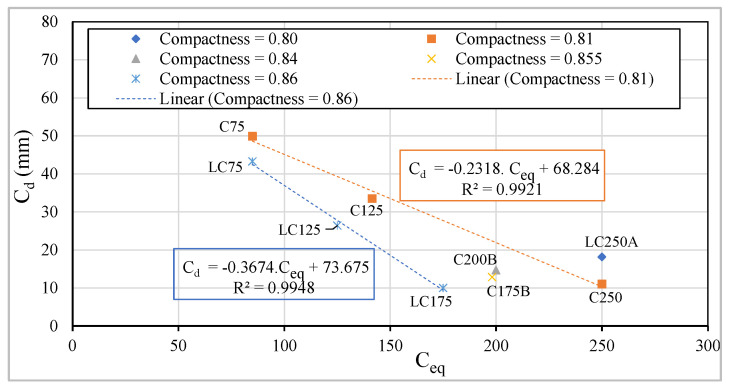
Relationship between C_d_ vs. C_eq._

Considering concretes with an equal dosage of cement, those in the LC series show lower carbonation depths than in the C series, due to the higher compactness of the first ones (Figure 18 and Figure 20), remembering that the LC series have only 250 kg/m^3^ of binder powder, being that dosage 350 kg/m^3^ in C series (Table 1). Maintaining the compactness, there is a higher linear but inverse correlation between the carbonation depth, C_d_, and the equivalent cement dosage (Figure 20).

Regarding the cement replacement partially by fly ash, the use of fly ash increases the carbonation speed, as can be confirmed by Figure 18, Figure 19 and Figure 20. The C75, LC75, C125 and C175B mixtures, which have fly ash in their formulation (Table 1) and present greater carbonation depths as higher is the amount of fly ash incorporated, contradicting the results of capillary absorption tests, which proved the beneficial effects of fly ash addition on reducing that property. Despite the carbonation test was performed after 28 days, it appears that the formation of crystals due to the pozzolanic effect is still in full development, since the pozzolanic reactions develop slowly. The structure will probably be more porous in concrete with fly ash and consequently, there is a greater ingress of carbon dioxide into the concrete. This may also be associated with the high dosage of fly ash used in the formulation as a partial substitute of cement [19,31,68,69].

According to Neville [19], the addition of fly ash to the concrete can cause two effects, a negative one, which is the decrease in calcium hydroxide that reacts with the pozzolanic silica present in the fly ash, thus making a smaller amount of CO_2_ necessary to consume all the remaining CH, thereby facilitating the decrease of pH. On the other hand, the positive effect is that from this reaction results in a denser structure in the hardened cement paste, which will hinder the diffusion of carbon dioxide and thus leads to slower carbonation speeds. Due to this situation, the dosage of fly ash is generally limited. The recommended proportions of cement replacement are of 0% to 40%, according to Teixeira et al. [31], as they argue that its use of large volumes presents some problems and one of them is the decrease of the pH concrete that leads to carbonation problems.

**Figure 21:**
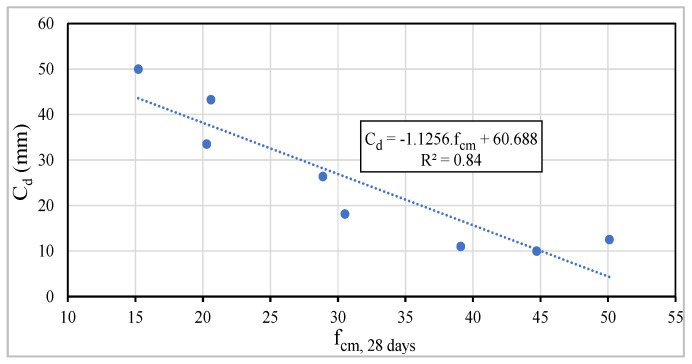
Relationship between carbonation depth after 42 days of exposure to CO_2_ and mechanical strength at 28 days.

The carbonation depth was also related to the concrete compressive strength at 28 days of age, as shown in Figure 21, noting a tendential linear correlation, being R^2^ equal to 0.84. This correlation is frequently analyzed in carbonation studies, but according to Neville [19], this approach is a gross, but debatable, simplification. Changes in the composition parameters can improve the mechanical properties, however, they can reduce the chemical durability.

Generally, it is concluded that it is possible to produce concrete with binder powder equal to 250 kg/m^3^, with only 175 kg/m^3^ of cement, and with high carbonation resistance.

### 4.6. Lifetime of Structures and Minimum Concrete Cover Regarding Carbonation

Based on the results obtained in carbonation test, it is possible to calculate the minimum concrete cover, c_min,dur_, to assure the required resistance against corrosion of the reinforcement during the intended service life for reinforced concrete structures (corrosion induced by carbonation) and to predict the service lifetime of the reinforced concrete structures using the developed concretes and the standard covers. Those calculations were based on the degradation model by corrosion of reinforcements, developed by Tuutti, and on the recommendations described on LNEC specification E465 [70]. Two types of structures were considered: (i) current structures with an intended service lifetime (t_g_) equal to 50 years, with a reliability class RC2 (structural class S4), which corresponds to a safety factor γ equal to 2.3; (ii) special structures with a t_g_ equal to 100-year-old, with a reliability class RC3 (structural class S5), which corresponds to a safety factor γ equal to 2.8.

To compute the minimum necessary cover, it is essential to calculate the carbonation resistance, RC65, of the studied concretes, to define the design period of initiation, tic, and, finally, to calculate the carbonation depth for various environmental exposure classes XC. Therefore, based on the experimental carbonation depth values, C_di_, at several days of exposure to carbon dioxide, with a concentration, C_acel_, approximately equal to 90 × 10^−3^ kg/m^3^, the carbonation resistance, R_C65_ (kg·year/m^5^), was determined using Equation (12). The results are shown in Table 4.

(12) $$R_{C65}=\frac{{2\times C}_{\mathrm{acel}}{\times t}_{i}}{C_{\mathrm{di}}^{2}}$$

The design period of initiation, t_ic_, is the difference between the intended service lifetime for the reinforced concrete structure, t_g_, and the propagation time, t_p_, affected by the safety factor mentioned above, see Equation (13). The propagation time depends of the environmental exposure class and was considered the minimum accordingly to the specification of LNEC E465 (Table 5).

(13) $$t_{\mathrm{ic}}{=\gamma (t}_{g}{-t}_{p})$$

**Figure 22:**
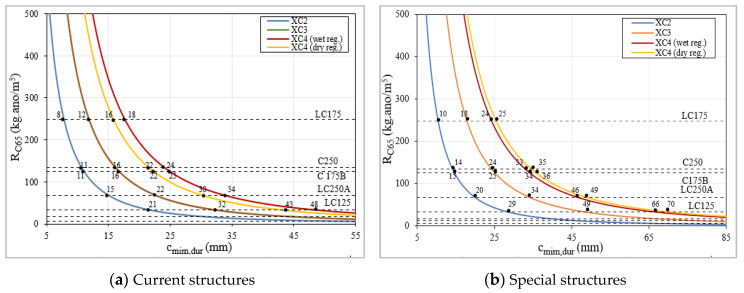
Minimum cover, c_min,dur_, regarding carbonation.

The minimum cover required to assure the resistance against corrosion of the reinforcement, c_min,dur_, is determined using Equation (14) and considering a time equal to t_ic_ (Table 6 and Figure 22). The result is the carbonation depth, C_di_, for those conditions, which corresponds to the c_min,dur_, k_0_ is a factor related to the test conditions and is equal to 3, t_0_ is the reference period and is equal to 1 year, k_2_ is related to concrete curing and assumes a value of 1, the parameters k_1_ and n, range between 0.2 and 0.41, and between 0.09 and 0.18, respectively.

(14) $$C_{\mathrm{di}}=\sqrt{\frac{2\;\times \;0.0007{\;\times \;t}_{\mathrm{ic}}}{R_{C65}}}\;\times \;\sqrt{k_{0}{\;\times \;k}_{1}{\;\times \;k}_{2}}\;\times \;{\left(\frac{t_{0}}{t_{\mathrm{ic}}}\right)}^{n}$$

The minimum cover, c_min,dur_, using some concretes are impractical, in particular, structures and structures located in environmental exposure class XC4. The LC75, C75, LC125 and C125 concretes require, in most cases, a minimum cover higher than the minimum value recommended by EC2, with differences ranging between 5 and 84 mm, for current structures, and between 10 and 128 mm, for special structures, depending on the environmental exposures’ classes. These results show that those concretes are not appropriate, or in certain cases are not the best option, to provide the proper protection against corrosion. On the other hand, the LC175, C175B, LC250, C200B and C250 concretes provide the proper resistance regarding carbonation, and in a high number of situations, have a higher resistance than that considered in EC2, since the minimum covers required are lower than those presented in the code, achieving a difference up to 17 mm.

**Figure 23:**
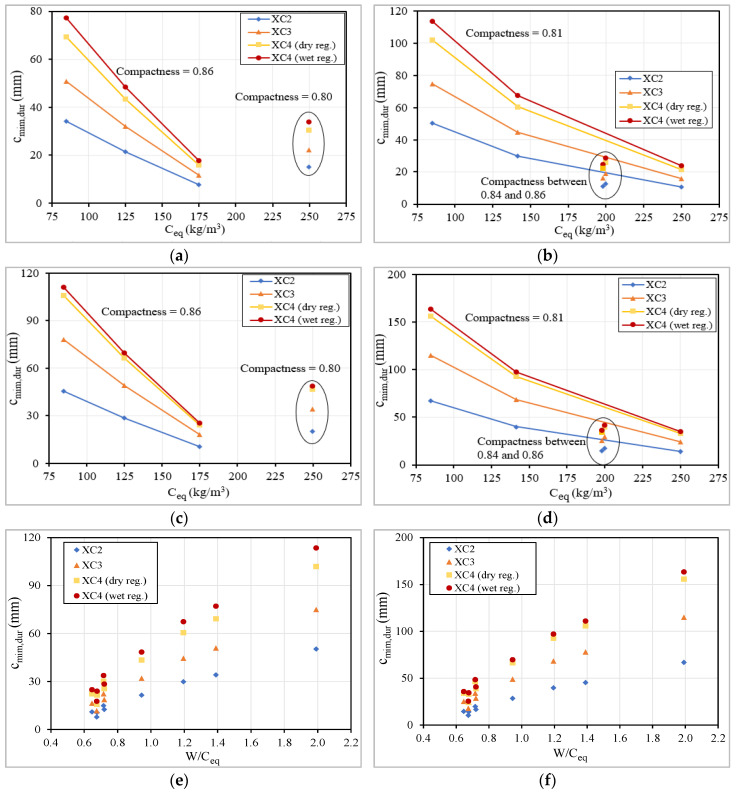
Relation between the c_min,dur_ vs. dosage of C_eq_ and between c_min,dur_ vs. W/C_eq_.:(**a**) current structures, total powder 250 kg/m^3^; (**b**) current structures, total powder 350 kg/m^3^; (**c**) special structures, total powder 250 kg/m^3^; (**d**) special structures, total powder 350 kg/m^3^; (**e**) current structures; (**f**) special structures.

The results show that it is necessary to use a minimum quantity of cement to assure that concrete develops the proper resistance against carbonation (Figure 23). To better understand this issue is also important to take into consideration the effect of fly ash additions used in the mixtures, additions with pozzolanic effect. It was recorded that an increase of equivalent dosage of cement above 200 kg/m^3^ does not have a significant influence on the carbonation resistance if mixtures have a relatively low compactness (Figure 23b,d). In this analysis, it is quite evident the positive influence of increased compactness, because a lower compactness implies a higher concrete cover, even if a higher dosage of cement is used. Using mixtures with a 350 kg/m^3^ of total powder (C series) is possible to slightly reduce the required compactness to achieve a similar carbonation resistance, comparatively with the produced concretes with 250 kg/m^3^ of total powder (LC series), because higher quantities of powder improve the workability, which may reduce the porosity of the matrix [64].

**Figure 24:**
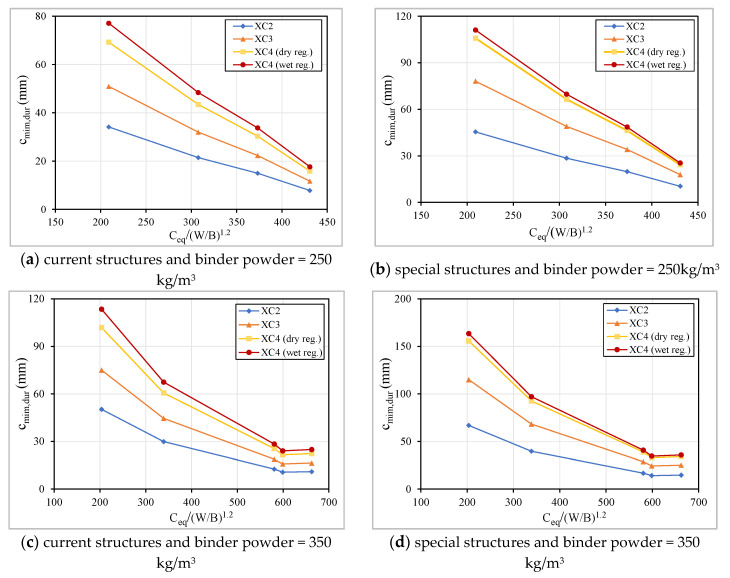
Relation between the c_min,dur_ vs. the relation C_eq_/(W/B)^1.2^.

It is important to mention that the concretes with low cement dosage are newly developed and, as such, do not follow the requirements regarding the dosages of binder and additions of the standard EN 206 (Table 7). The W/C_eq_ ratio reflects both effects analyzed above, compactness and equivalent dosage of cement, in one parameter. This ratio was related with c_min,dur_ and it is clear that a ratio above 0.72 increases significantly the concrete cover required to protect the reinforcement steel (Figure 23). This limit is a slightly higher than those recommended in EN 206 (Table 7), meaning that there is a margin for optimization if the constituents and the distribution of the particles size were properly defined. The correlation presented in Figure 24 also allows both effects to be considered, the compactness (that is inversely related with W/B ratio) and the equivalent cement dosage.

For an overall analysis, where sustainability is taken into consideration, it should be noted that the use of LC175 allows to use a concrete cover below the minimum standard, almost in all cases, and additionally it contains only 175 kg/m^3^ of cement, which is also lower than the minimum standard recommendation of 280 kg/m^3^ for XC2 and XC3 and 300 kg/m^3^ for XC4. So, the quantity of cement can be reduced by between 37.5% to 42%, depending on the environmental exposure classes XC. These results are very important because they have significant impact on the structures cost and simultaneously demonstrate that this concrete is clearly eco-friendlier.

The prediction of the possible service lifetime for reinforced concrete structures produced using the developed concretes and the covers recommended in the codes was carried out for several environmental conditions XC and for both types of structures S4 and S5. Equation (14) was used to determine the t_ic_ value, knowing the remaining variables, and in this case C_di_ was considered equal to the minimum cover presented in EC2. Knowing the t_ic_ and t_p_ values, the service lifetime, t_g_, was determined using Equation (13).

Analyzing the values in Table 8, the LC175, C175B, C200B and C250 concretes have a carbonation resistance, R_C65_, more than enough for the exposure class XC, having a margin to decrease the minimum cover used in reinforced concrete structures. On XC2 conditions, the service lifetime values are quite high because the carbonation depth, C_d_, is very low in terms of environmental conditions. For special structures and under XC4 in wet regime conditions, only the LC175 and C250 presents a proper performance, since they are the only ones with a predicted service lifetime higher than 100 years.

These results also show that concretes with very low dosages of cement, like LC75, C75, LC125 and C125, are not appropriate to provide proper protection against corrosion under XC exposure conditions. It can also be stated, comparing the LC250A with C250, but that is not only the equivalent dosage of cement that matters concerning this resistance, it is also very important to use in this type of concrete a limestone filler to ensure a certain quantity of powder in the mixtures, which must be around 350 kg/m^3^. Nevertheless, it is generally possible to produce concretes with low-carbon cements that provide a proper performance against carbonation and, consequently, a long service life, which is a major step to achieve a more sustainable construction sector.

The possible addition of fibers into LCC may be an important future research topic to improve the carbon reduction and its sustainability even more. The bond strength between steel rebars and low cement concrete is significantly improved by the higher compactness of LCC [71]; thus, it is expected that similar influence might occur on bond strength between the matrix and the reinforcement fibers. However, major challenges are expected in the development of fiber reinforced LCC, since rigid steel fibers have a high influence on the aggregate packing [72] and high volumes of paste are usually necessary [73] to obtain proper rheology and workability. To overcome those expected difficulties, the powder and paste volumes must be probably increased, by adding limestone filler, or limited fiber dosage must be used. Nevertheless, when combined with the addition of fibers, the formulation parameters of LCC, including the compactness and the binder dosage, may promote higher reduction of cement proportion and potential improvements of mechanical and durability performances, and are thus more sustainable.

## 5. Conclusions

Based on results analysis of the experimental study of concretes with low cement content (LCC), developed by varying the dosage of binder, the compactness, the rate of cement replacement by additions and the granulometric reference curve, it was possible to draw the following conclusions.

- Shrinkage: (i) LCC concrete with high compactness of 0.86 and reduced powder dosage (250 kg/m^3^) promotes reduced shrinkage, tending to increase with the increase of cement dosage; however, in concrete with powder dosage of 350 kg/m^3^, the increase of compactness tends to gradually decrease shrinkage; (ii) due to the inadequacy of the EC2 prediction of shrinkage, compared to experimental values, a correction is proposed to improve the curves development, where a β_shape_ coefficient assumes different values (0.06 for concrete with powder of 350 kg/m^3^ and 0.08 for concrete with powder of 250 kg/m^3^); (iii) beyond the parameters considered by the concrete codes, the concrete compactness has a noticeable influence on the amplitude of concrete shrinkage, mainly when combined with reduced binder powder (250 kg/m^3^) and very reduced cement dosage, where the experimental/prediction ratio of shrinkage can go down to 0.4.
- Creep: (i) the granulometric reference curve (Faury vs. Alfred), in concrete with reduced binder powder (250 kg/m^3^), does not have a relevant influence on the evolution and amplitude of creep coefficient; (ii) similarly to the known behavior of current concrete, the creep coefficient is highly influenced by the concrete strength; thus, LCC presents reduction of creep coefficient for concrete with high compactness (0.86) and higher cement dosage (175 kg/m^3^), which has also higher strength; (iii) a divergence between the shape of the creep curve experimentally obtained and that according to EC2 is noticeable; however, the shape of the creep curve can be improved significantly by adjusting the α_t_ coefficient on β_c_ (t,t_0_) parameter, from 0.30 to 0.15, for LCC; (iv) there is a huge difference between experimental and EC2 predicted values for the creep of LCC, mainly in concretes with lower cement dosage and with lower compactness (0.80 to 0.81), thus with higher W/B ratio, the creep being ratio experimental/code around 0.3; (v) W/C_eq_ ratio has a significant influence on that difference, therefore, a corrective parameter is proposed to be included on the β (f_cm_) coefficient of EC2 to significantly improve the code prediction, namely $\beta_{\mathrm{Ceq}}=2.5\;\times \;{\left(W/\mathrm{Ceq}\right)}^{1.5}$, which was obtained by correlation analysis.
- Water absorption by immersion: (i) the increase of compactness has a great influence on the reduction of water absorption, since the values for concretes with lower compactness (0.80 to 0.81) are close to 15% and those values reduce to below 10% for concretes with compactness of 0.86; (ii) the W/C_eq_ ratio also has an influence on absorption, although it is less significant; thus, the relation that incorporates compactness and that ratio, (W/C_eq_)^0.2^/Compactness^6^, proved by correlation analysis, has high influence on water absorption.
- Water absorption by capillarity: (i) high compactness of LCC combined with higher cement dosage significantly reduces the capillary absorption; (ii) fly ash addition as partial replacement of cement also promotes a significant decrease in capillary absorption; however, its excessive dosage may jeopardize the concrete performance regarding capillarity; (iii) the capillary coefficients of the LCC characterized allow all concretes to be classified as high quality, except the one with reduced compactness (0.81) and very reduced cement dosage (75 kg/m^3^).
- Carbonation resistance: (i) maintaining the cement dosage, compactness increase has a significant influence on reducing carbonation depth, since the LC series (concrete with high compactness of 0.86 and with 250 kg/m^3^ of binder powder) exhibits much lower carbonation depth than the corresponding C series (concrete with lower compactness and 350 kg/m^3^ of binder powder); (ii) maintaining the binder dosage, the carbonation depth decreases with the increase of cement or equivalent cement dosages, since it reduces the W/C_eq_ ratio; (iii) the higher the amount of fly ash incorporated in the concrete, replacing cement dosage, the lower the carbonation resistance, because it increases the carbonation velocity; (iv) it is possible to produce concrete with good structural performance, with low binder powder of 250 kg/m^3^ and only 175 kg/m^3^ of cement dosage, developing higher carbonation resistance, in circa 10%, than current formulation concrete with 250 kg/m^3^ of cement dosage and binder powder of 350 kg/m^3^.
- Minimum cover required to avoid corrosion induced by carbonation: (i) it is necessary to use a minimum cement dosage, since only concretes with cement dosage equal or higher than 175 kg/m^3^ (even though with very different formulation parameters) have adequate resistance to carbonation for general exposure; for those concretes the minimum required covers are lower than those presented in EC2, reaching differences of up to 17 mm; (ii) the compactness increase has also high influence on reducing the minimum cover, since it increases the concrete strength and carbonation resistance; reducing the concrete compactness implies increasing the cover, even if higher cement dosage is used; the LCC with 175 kg/m^3^ of cement and compactness of 0.86 presents much higher resistance to carbonation compared to that of all formulated concrete, including those with equivalent cement dosage between 200 and 250 kg/m^3^.
- Lifetime of structures due to carbonation exposure: (i) higher cement dosages promotes longer lifetime; concretes with at least 175 kg/m^3^ of cement dosage have, submitted to XC2 and XC3 conditions, service lifetime values above the minimum, for current (50 years) and special (100 years) structures and, for XC2, the values are quite high; (ii) for special structures and under XC4 in wet conditions, only the LC175 and C250 have adequate performance, the first due to the high compactness and the latter due to higher cement dosage, being the only mixtures with an expected lifetime of more than 100 years.
- LCC mixtures with a good performance and a long lifetime: (i) concrete with at least 175 kg/m^3^ of cement dosage reveals adequate combined mechanical, time-dependent and durability performances; (ii) concrete LC175 (with high compactness of 0.86 and powder dosage of only 250 kg/m^3^) is revealed to be the most eco-efficient of the concretes studied herein, since for current and special structures, and for any type of XC exposure, it is possible to reduce the cover below the standard minimum, presenting a long lifetime; (iii) the amount of cement can be reduced between 37.5% and 42%, depending on the environmental exposure classes XC, comparing the LCC concrete which contains only 175 kg/m^3^ of cement with the minimum recommendation of 280 kg/m^3^, for XC2 and XC3, and 300 kg/m^3^ for XC4.

## Figures and Tables

**Table 1:** Dosages of constituents and formulation parameters of the studied concretes.

Constituents	LC75	LC75F	C75	LC125	LC125F	C125	LC175	LC175F	C175B	C200B	LC250A	C250
C (kg/m^3^)	75	75	75	125	125	125	175	175	175	200	250	250
Lf (kg/m^3^)	75	75	75	125	125	125	75	75	100	150	-	100
FA (kg/m^3^)	100	100	200	-	-	100	-	-	75	-	-	-
SKY (kg/m^3^)	2.3	2.3	0.7	2.5	2.5	0.8	2.6	2.6	2.1	1.0	0.8	1.0
W (kg/m^3^)	118	118	169	118	118	169	118	118	128	144	179	169
S0/3 (kg/m^3^)	44	587	286	44	671	371	44	747	181	186	218	492
S0/4 (kg/m^3^)	1068	308	585	1080	244	520	1084	210	762	742	817	427
G4/8 (kg/m^3^)	284	78	106	287	82	110	288	98	92	96	278	116
C6/14 (kg/m^3^)	623	1050	798	631	1049	797	633	997	894	869	587	795
Total aggregates (kg/m^3^)	2018	2023	1774	2042	2047	1798	2048	2053	1929	1893	1900	1831
W/C	1.57	1.57	2.26	0.94	0.94	1.35	0.67	0.67	0.73	0.72	0.72	0.68
Equiv.-cement, C_eq_ (kg/m^3^)	85	85	85	125	125	142	175	175	198	200	250	250
W/C_eq_	1.39	1.39	1.99	0.94	0.94	1.19	0.67	0.67	0.65	0.72	0.72	0.68
W/B	0.47	0.47	0.48	0.47	0.47	0.48	0.47	0.47	0.37	0.41	0.72	0.48
Compactness	0.86	0.86	0.81	0.86	0.86	0.81	0.86	0.86	0.86	0.84	0.80	0.81

**Table 2:** Mechanical properties of studied concretes.

LCC Mixtures	f_cm,7_ (MPa)	f_cm,28_ (MPa)	f_cm,56_ (MPa)	E_cm_ (GPa)	f_ctm,f_ (MPa)	f_ctm,sp_ (MPa)	Slump (mm)	D_Comp
LC75	11.6	20.6	26.5	37.4	3.2	1.5	-	1.21
LC75F	15.7	22.1	-	28.7	-	-	50	-
C75	7.6	15.2	20.5	-	2.1	0.7	90	-
LC125	25.2	28.9	33.9	40.6	4.5	2.3	-	1.23
LC125F	26.4	29.4	-	34.5	-	-	45	-
C125	14.3	20.3	26.5	-	3.6	1.5	120	-
LC175	35.0	44.7	50.2	42.4	6.9	3.4	-	1.25
LC175F	36.8	45.9	-	38.2	-	-	65	-
C175B	36.2	50.1	52.9	47.9	5.7	3.2	107	-
C200B	32.9	38.2	39.1	41.3	5.8	3.0	110	-
LC250A	25.8	30.5	34.3	38.1	5.1	2.2	-	1.16
C250	34.9	39.1	41.5	36.5	5.4	2.9	80	-

**Table 3:** Results of carbonation depth, C_d_ (mm).

C_d_ (mm)					
LCC Mixtures	Days				
	3	7	14	28	42
LC75	10.0	15.0	23.3	36.5	43.3
C75	17.5	22.5	39.0	49.9	-
LC125	5.5	11.0	15.3	21.8	26.4
C125	10.0	15.5	20.0	27.8	33.5
LC175	2.0	3.5	5.8	9.0	10.0
C175B	-	4.8	-	10.3	12.9
C200B	-	5.3	-	12.3	14.7
LC250A	4.5	6.5	10.3	15.8	18.1
C250	3.5	5.0	8.3	10.0	11.0

**Table 4:** The carbonation resistance, R_C65_.

R_C65_ (kg·year/m^5^)						
LCC Mixtures	-	-	Days	-	-	Average
	3	7	14	28	42	
LC75	15	15	13	10	11	13
C75	5	7	5	6	-	5
LC125	49	29	30	29	30	33
C125	15	14	17	18	18	17
LC175	370	282	209	170	207	248
C175B	-	153	-	129	125	125
LC250A	73	82	66	56	63	68
C200B	-	123	-	91	15	80
C250	121	138	101	138	171	134

**Table 5:** Minimum propagation period and the initiation period due to carbonation.

Intended Service Lifetime	Propagation Time and Period of Initiation	XC2 (Wet, Rarely Dry)	XC3 (Moderate Humidity)	XC4 (Dry Regime)	XC4 (Wet Regime)
t_g_ = 50 years (RC2)	t_p_ (years)	10	45	15	5
	t_ic_ (years)	92	12	80	105
t_g_ = 100 years (RC3)	t_p_ (years)	20	90	20	10
	t_ic_ (years)	224	28	224	252

**Table 6:** Minimum cover according to standards and for the studied concrete regarding carbonation.

Minimum Cover c_min,dur_ (mm)								
Structural Class	Current Structures (Class S4)				Special Structures (Class S5)			
Exposure Classes	XC2	XC3	XC4 (Dry Reg.)	XC4(Wet Reg.)	XC2	XC3	XC4 (Dry Reg.)	XC4 (Wet Reg.)
EC2	25	25	30		30	30	35	
LC75	34	51	69	77	45	78	106	111
C75	50	75	102	114	67	115	156	163
LC125	21	32	43	48	29	49	66	70
C125	30	45	61	67	40	68	93	97
LC175	8	12	16	18	10	18	24	25
C175B	11	16	22	25	15	25	34	36
LC250A	15	22	30	34	20	34	46	49
C200B	13	19	25	28	17	29	39	41
C250	11	16	22	24	14	24	33	35

**Table 7:** Minimum dosage of cement according to standards and equivalent dosages used in the concretes studied.

Structural Class			Current and Special Structures (Classes S4 and S5)		
Type of Cement			CEM I; CEM II/A (1)		
Exposure Class			XC2	XC3	XC4 (Wet and Dry Reg.)
EN 206	Minimum dosage of C (kg/m^3^)		280	280	300
	Maximum W/C ratio		0.60	0.55	0.50
-	-		Total powder (kg/m^3^)	Equivalent dosage of cement, C_eq_ (kg/m^3^)	W/C_eq_
-	Concretes	LC75	250	85	1.39
		LC125		125	0.94
		LC175		175	0.67
		LC250A		250	0.47
		C75	350	85	2.25
		C125		142	0.97
		C175B		198	0.73
		C200B		200	0.72
		C250		250	0.68

**Table 8:** Service lifetime (years) for current and special structures produced with the studied concrete under environmental conditions XC.

Structural Class		Current Structures (Class S4, RC2, 50 Years)				Special Structures (Class S5, RC3, 100 Years)			
Exposure Class		XC2	XC3	XC4 (Dry Reg.)	XC4 (Wet Reg.)	XC2	XC3	XC4 (Dry Reg.)	XC4 (Wet Reg.)
Concretes	c_min,dur_ (mm)	25		30		30		35	
LC75	t_g_ (years)	25	46	19	9	42	91	25	15
C75		15	46	17	7	27	91	22	12
LC125		75	48	29	19	114	94	37	27
C125		33	47	21	11	53	92	27	17
LC175		>>>> 200	69	179	169	>> 200	119	216	206
C175B		>> 200	57	86	76	>> 200	104	105	95
LC250A		>> 200	51	49	39	>> 200	98	60	50
C200B		>> 200	54	67	57	>> 200	101	82	72
C250		>> 200	58	92	82	>> 200	105	113	103
